# Gepants: targeting the CGRP pathway for migraine relief

**DOI:** 10.3389/fphar.2025.1708226

**Published:** 2025-11-21

**Authors:** Bernadetta Jakubowska, Magdalena Sowa-Kućma

**Affiliations:** 1 Student’s Science Club of Physiology “NEURON”, Faculty of Medicine, University of Rzeszów, Rzeszów, Poland; 2 Department of Human Physiology, Faculty of Medicine, University of Rzeszów, Rzeszów, Poland; 3 Centre for Innovative Research in Medical and Natural Sciences, Collegium Medicum, University of Rzeszów, Rzeszów, Poland

**Keywords:** gepants, CGRP receptor antagonists, migraine, atogepant, ubrogepant, rimegepant, zavegepan

## Abstract

**Background:**

Calcitonin gene–related peptide (CGRP) receptor antagonist (collectively known as gepant) have emerged as effective therapies for both acute migraine treatment and preventive management. Several new agents (atogepant, ubrogepant, rimegepant, and zavegepant) expand therapeutic options beyond traditional triptans and monoclonal antibodies.

**Objective:**

To summarize and critically evaluate the pharmacology, clinical efficacy, safety, and positioning of currently approved gepants for migraine treatment and prevention.

**Methods:**

A structured literature search was performed in PubMed/MEDLINE, Embase, Web of Science, Scopus, Cochrane Library, and ClinicalTrials.gov for studies published from 1998 to September 2025. Search terms included “gepant,” “CGRP receptor antagonist,” “atogepant,” “ubrogepant,” “rimegepant,” “zavegepant,” “pharmacokinetics,” “efficacy,” and “safety,” combined with Boolean operators. Peer-reviewed clinical studies providing data on pharmacology, efficacy, or safety were included; case reports, conference abstracts, and non-English articles were excluded.

**Results:**

Four gepants are currently approved for clinical use. Atogepant is indicated solely for prevention, while ubrogepant and zavegepant are approved for acute treatment; rimegepant is approved for both indications. All agents exhibit favorable pharmacokinetic profiles, with oral or intranasal formulations enabling rapid onset and convenient dosing. Across randomized controlled trials, gepants demonstrated significant reductions in monthly migraine days for prevention and high rates of 2-h pain freedom in acute treatment compared with placebo. Safety profiles were generally benign, with mild adverse events such as nausea and fatigue and no evidence of vasoconstriction or hepatotoxicity in contemporary studies.

**Conclusion:**

Gepants represent a valuable advancement in migraine therapy, offering effective acute and preventive options with favorable tolerability, particularly for patients who cannot use triptans or who have cardiovascular risk factors. Ongoing studies will clarify long-term safety, real-world effectiveness, and potential roles in combination or sequential therapy.

## 1 Introduction

Migraine is a highly prevalent neurological disorder characterized by recurrent, often severe headaches accompanied by nausea, vomiting, and hypersensitivity to light and sound. It affects nearly one billion individuals worldwide, with a disproportionate burden on women after puberty (GBD, 2016 Headache Collaborators, 2018; Dong et al., 2025; Steiner et al., 2020). Beyond the immediate pain, migraine has profound consequences on health-related quality of life (HRQoL), daily functioning, and psychosocial wellbeing. Studies have shown that migraine sufferers experience impairments across mobility, self-care, work productivity, and mental health, including a higher risk of depression and suicidal behavior (Dimitrz and Golicki, 2021; Vetvik and MacGregor, 2017; Neumeier et al., 2022). The disorder also leads to substantial societal and economic costs through absenteeism, presenteeism, and increased healthcare utilization (Steiner et al., 2020). These factors underscore the urgent need for effective and well-tolerated treatments targeting the underlying pathophysiology of migraine.

Pharmacological management of migraine has evolved considerably over the past century. In the 19th century, ergot alkaloids such as ergotamine and dihydroergotamine were the first empirically used antimigraine agents, later potentiated with caffeine (Olesen et al., 2023). Their anti-serotonergic effects were confirmed in the mid-20th century, when Sicuteri demonstrated the efficacy of methysergide, a semisynthetic ergot derivative (Koehler and Tfelt-Hansen, 2008). However, the pleiotropic receptor activity of ergot alkaloids, including actions on adrenergic and dopaminergic receptors, was associated with a wide range of adverse effects, such as insomnia, gastrointestinal complaints, arrhythmias, and vascular complications (Silberstein and McCrory, 2003). The recognition that ergot alkaloids exert agonistic effects on 5-HT1B and 5-HT1D receptors led to the development of more selective drugs - the triptans (Edvinsson et al., 2022; Whealy and Becker, 2024). These agents, now considered the standard of care for acute migraine, act by activating vascular 5-HT1B receptors, inducing cranial vasoconstriction, and presynaptic 5-HT1D receptors, thereby inhibiting trigeminovascular activation (Edvinsson et al., 2022; Whealy and Becker, 2024). In addition, most triptans display affinity for 5-HT1F receptors, a property that has informed the development of newer, more selective antimigraine therapies such as lasmiditan, which targets 5-HT1F exclusively and avoids vasoconstrictive mechanisms (Rubio-Beltrán et al., 2018; Vila-Pueyo et al., 2022). While triptans are effective in a significant proportion of patients, their vasoconstrictive mechanism contraindicates their use in individuals with cardiovascular risk (Tfelt-Han et al., 2000). Moreover, approximately one-third of patients achieve complete pain freedom within 2 h, while 30%–40% experience recurrence (de Boer et al., 2023). Non-specific analgesics, such as NSAIDs, are often combined with triptans to achieve synergistic effects, yet many patients remain insufficiently controlled (de Boer et al., 2023). Preventive therapy has likewise expanded from the use of antiepileptic drugs (e.g., topiramate, valproate), antidepressants (e.g., amitriptyline), and beta-blockers (e.g., propranolol, metoprolol) to more targeted options such as onabotulinumtoxin. A for chronic migraine (Dodick et al., 2010; American Headache Society, 2019; Duman et al., 2021; Edvinsson, 2017; Silvestro et al., 2023). Although effective, these treatments are often limited by tolerability, adverse effects, or contraindications, leaving an unmet need for safer and more specific therapeutic options.

A major breakthrough in understanding migraine pathophysiology has been the identification of calcitonin gene–related peptide (CGRP) as a key mediator within the trigeminovascular system. CGRP contributes to neurogenic inflammation, vasodilation, and modulation of nociceptive transmission within the trigeminovascular system (Edvinsson, 2017; Silvestro et al., 2023). This insight gave rise to the development of CGRP-targeted therapies, including monoclonal antibodies and small-molecule CGRP receptor antagonists (known as the gepants). The history of gepants spans three generations. First-generation agents, such as olcegepant and telcagepant, demonstrated efficacy but were discontinued due to intravenous-only administration (olcegepant) or hepatotoxicity (telcagepant) (Olesen et al., 2004; Ho et al., 2008). Second-generation gepants—including ubrogepant, rimegepant, and atogepant—overcame these limitations, showing favorable safety and efficacy (Dodick et al., 2019; Croop et al., 2019a; Goadsby et al., 2020). Ubrogepant and rimegepant are approved for the acute treatment of migraine, while rimegepant and atogepant have also been approved for prevention (U.S. Food and Drug Administration, 2021). Most recently, third-generation agents have emerged, exemplified by zavegepant, the first intranasal gepant, which provides a novel, rapid-onset, non-oral route of administration (Lipton et al., 2023a).

In conclusion, the progressive refinement of migraine pharmacotherapy, from ergot alkaloids to triptans and now to gepants, reflects a shift toward receptor-specific, mechanism-based treatments. Gepants represent a promising advance by directly targeting the CGRP pathway, offering both acute and preventive efficacy with a favorable safety profile. This review aims to synthesize current knowledge and critically examine the role of gepants in the treatment and prevention of migraine, highlighting their pharmacological evolution from first- to third-generation agents. A structured search was conducted in PubMed/MEDLINE, Embase, Web of Science, Scopus, the Cochrane Library, and ClinicalTrials.gov for studies published from 1998 through September 2025 on small-molecule CGRP receptor antagonists (gepants). Search terms included “gepant”, “CGRP receptor antagonist”, “calcitonin gene-related peptide”, “atogepant”, “ubrogepant”, “rimegepant”, “zavegepant”, “olcegepant”, “telcagepant”, “pharmacokinetics”, “pharmacodynamics”, “CYP3A4”, “efficacy”, “safety”, “acute treatment”, “prevention”, and “clinical trial”, combined with Boolean operators. Reference lists of included papers and recent reviews were also screened. Eligible studies were peer-reviewed preclinical and clinical investigations reporting pharmacology, efficacy, or safety data. Case reports, conference abstracts without full reports, non–peer-reviewed sources, and non-english articles were excluded.

## 2 Migraine - scale of the problem, classification and its impact on quality of life

### 2.1 Epidemiology

According to the Global Burden of Disease 2019, migraine is the second leading cause of disability worldwide and the leading cause among young women, affecting approximately 14.4% of people globally. Between 1990 and 2019, there was a 40% global increase in migraine incidence, reaching nearly 88 million cases in 2019 (with an incidence rate of 1132.79 [95% UI: 990.45, 1275.02] per 100,000 people). In 2019 the total estimated number of migraine sufferers worldwide was approximately 1.1 billion (Fan et al., 2023). The annual increase in migraine incidence varied, ranging from 3.45% (95% CI: 2.38, 4.54) in regions with high and medium socio-demographic index (SDI) to −4.02% (95% CI: −4.79, −3.18) in low-SDI regions (Duman et al., 2021). Furthermore, migraine was among the top five causes of age-standardized years lived with disability (YLDs) in over 190 countries in 1990–2015. In 2016, migraine rose to second place in this ranking, which was due to the classification of medication-overuse headache (previously considered a separate disease entity) under the definition of migraine pain. Migraine was also included among the 369 diseases classified under disability-adjusted life years (DALY) for individuals of reproductive age. Migraine headaches affect 18.9% (18.1–19.7) of women and 9.8% (9.4–10.2) of men (Steiner et al., 2020). The prevalence of migraine does not differ significantly between boys and girls before puberty, ranging from 2% to 5% at ages 7–9% and 4%–5% at ages 10–12 (GBD 2016 Headache Collaborators, 2018). However, as individuals grow older, this pattern changes, with migraine frequency increasing more in girls. During pregnancy and menopause, migraines tend to subside. It is also worth noting that both the prevalence and the highest YLD rate for migraine occur in individuals aged 35–39 years (GBD 2019 Diseases and Injuries Collaborators, 2020).

### 2.2 Migraine attacks

**FIGURE 1:**
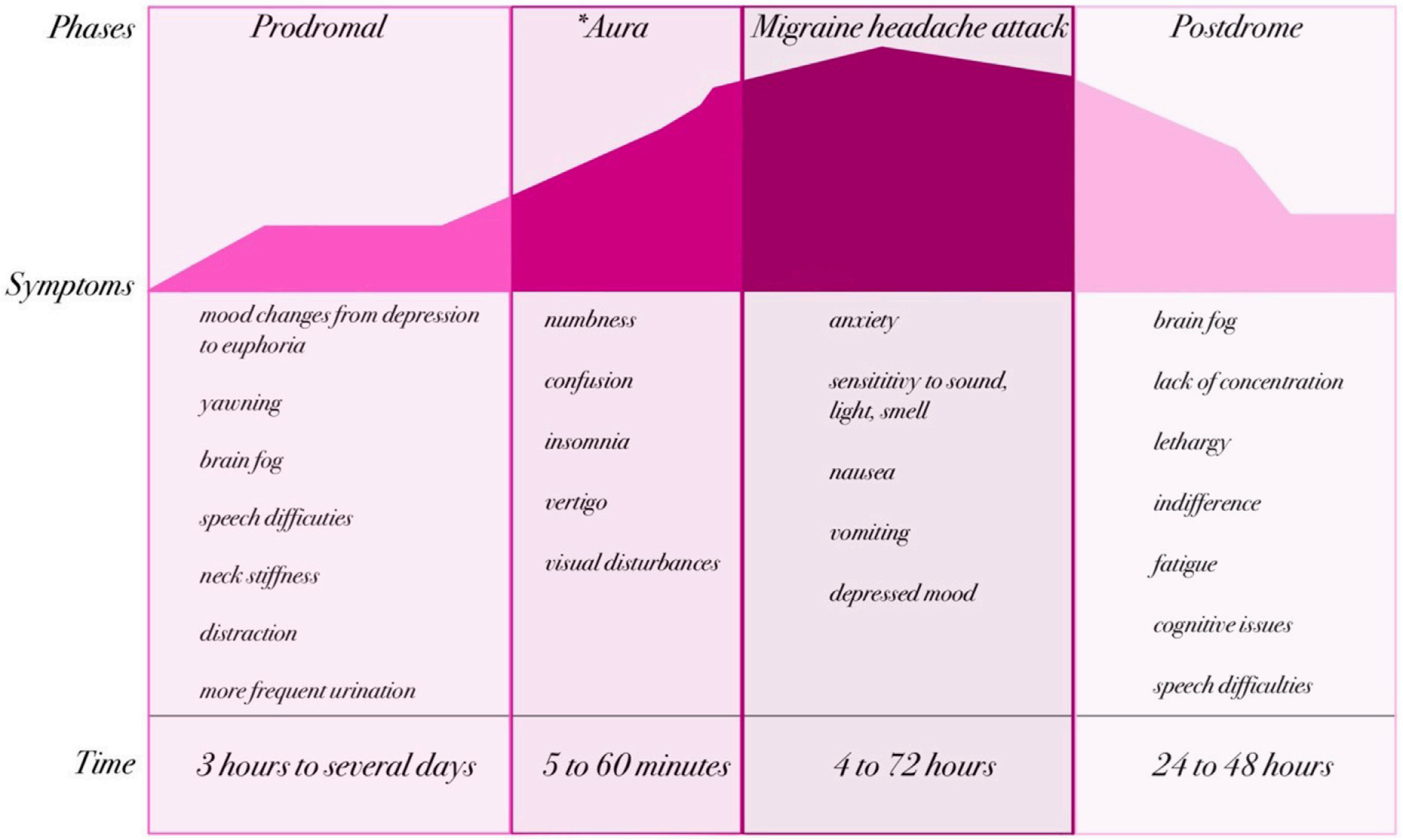
Phases and clinical manifestations of migraine (created by BioRender based on (GBD 2019 Diseases and Injuries Collaborators, 2020; Headache Classification Committee of the International Headache Society IHS, 2018)). The natural course of a migraine attack can be divided into up to four phases: prodromal, aura (*present in approximately one-third of patients), headache, and postdromal. Each phase is characterized by specific neurological and systemic symptoms that evolve over time. The prodrome may last from several hours to days and includes symptoms such as mood changes, yawning, or neck stiffness. When present, the aura typically lasts 5–60 min and is characterized by transient neurological disturbances such as visual or sensory changes. The migraine headache phase lasts from 4 to 72 h and involves severe headache accompanied by nausea, vomiting, and hypersensitivity to sensory stimuli. Finally, the postdrome phase (24–48 h) is often marked by fatigue, cognitive impairment, and residual discomfort.

Migraine is characterized by recurring attacks—most often described as episodic, unilateral, pulsating headaches of moderate to severe intensity (Headache Classification Committee of the International Headache Society IHS, 2018). These attacks typically last from 4 to 72 h and are commonly accompanied by nausea (sometimes with vomiting), visual and speech disturbances, photophobia, phonophobia, movement intolerance, or difficulties in concentration (Steiner et al., 2020). A migraine attack is conventionally divided into four phases: the prodromal (premonitory) phase, aura (when present), headache (often with accompanying symptoms), and postdromal (postdrome) phase (Figure 1). It is important to emphasize that aura occurs in only about one-third of migraine patients (GBD 2016 Headache Collaborators, 2018; Dong et al., 2025), and thus the classic four-phase schema does not apply universally. Some authors also describe an additional “resolution” interval between the headache and postdrome phases, often occurring during sleep; however, this is more commonly treated as part of the postdromal phase in the published literature (Goadsby et al., 2017; Charles, 2018).

Migraine headaches are classified as primary headaches, meaning the pain is not secondary to another disorder. Prodromal symptoms may include nausea, photophobia, phonophobia, neck stiffness, drowsiness, yawning, and pallor. Notably, these prodromal signs can precede the headache by up to 72 h, reflecting early changes within the central nervous system (CNS). These symptoms are typically non-nociceptive, pointing to a cerebral (rather than peripheral nociceptive) origin (Kelman, 2004; Schoonman et al., 2006; Qubty and Patniyot, 2020). Aura may present with visual disturbances, sensory phenomena, speech or language difficulties, or motor symptoms. These neurological symptoms are transient and generally resolve within 5–60 min (GBD 2016 Headache Collaborators, 2018; Dong et al., 2025). In addition to the canonical phases, many patients experience nonspecific symptoms—such as fatigue, cognitive slowing, or insomnia—at various points in the migraine cycle, blurring the boundaries between phases. Moreover, the chronological progression of migraine phases is not always strictly linear: phases may overlap, skip, or merge. For instance, between the aura and headache phases there may be a brief symptom-free interval of up to 60 min (Headache Classification Committee of the International Headache Society IHS, 2018; Cutrer and Huerter, 2007). The headache phase is marked by pain that worsens with physical activity and may last from 4 to 72 h (Goadsby et al., 2017). During the postdromal phase—the period following headache resolution—patients often report lingering symptoms such as fatigue, drowsiness, impaired cognition, speech difficulty, apathy, or confusion, all of which can significantly impact daily functioning (Lipton et al., 2023b; Kelman, 2006).

### 2.3 Classification

In 1988, the International Classification of Headache Disorders (ICHD-1) published the first diagnostic criteria for migraine, which have been gradually modified over the past three decades. Currently, specialists follow the latest revision from 2018 (International Classification of Headache Disorders, 3rd edition; ICHD-3), which distinguishes several clinical forms of migraine (Headache Classification Committee of the International Headache Society IHS, 2018; International Headache Society, 2025). These include migraine with aura, accounting for approximately 15% of cases, and the more common migraine without aura, affecting about 85% of patients (Lucas, 2021). Based on the frequency of migraine attacks, migraines are classified as episodic migraine, where headaches occur less than 15 days per month, and chronic migraine, which is defined as headaches occurring on ≥15 days per month for at least 3 months. An intermediate category, transformed migraine, describes the transition from prolonged episodic migraine to chronic migraine (Mungoven et al., 2021). Among the key classifications outlined in ICHD-3 (Headache Classification Committee of the International Headache Society IHS, 2018; International Headache Society, 2025), menstrual migraine is characterized by migraine attacks occurring on the second or third day of menstruation in at least two-thirds of menstrual cycles. Vestibular migraine, on the other hand, is associated with visual aura, photophobia, phonophobia, and at least two of four migraine headache characteristics: unilateral location, pulsating nature, moderate to severe intensity, and worsening with movement or avoidance of physical activity. Migraine diagnosis is primarily based on medical history and symptoms. The classification of migraine has significant clinical implications for pharmacotherapy, as different types of migraines require distinct treatment approaches (Verhagen et al., 2022).

### 2.4 Etiopathogenesis

Migraine is now recognized as a complex neurovascular disorder involving both peripheral and central mechanisms. Despite decades of research, the underlying pathophysiology remains only partially understood. Early hypotheses focused largely on vascular changes, beginning with the landmark work of Graham and Wolff in 1938, who proposed that abnormal dilation of cerebral vessels was the key driver of migraine pain (Goadsby et al., 2017). A major advance supporting the vascular theory came with the development of sumatriptan, the first tryptamine-based drug in the triptan class, introduced in the Netherlands in 1991 and in the United States in 1993. Sumatriptan constricts abnormally dilated intracranial arteries, restores normal blood flow, and blocks the release of pro-inflammatory neurotransmitters at perivascular nerve endings - actions mediated by selective binding to the 5-HT1D receptor (van den Broek et al., 2002). However, most triptans also display agonistic activity at 5-HT1F receptors, and this pharmacological profile has provided the rationale for the development of newer antimigraine agents such as lasmiditan, which selectively target 5-HT1F without inducing vasoconstriction (Rubio-Beltrán et al., 2018; Vila-Pueyo et al., 2022). The discovery that sumatriptan also influences neuronal pathways prompted a shift toward a broader view that incorporates neural mechanisms (Kogelman et al., 2023; Guo et al., 2024). Long before this, Edward Liveing had suggested that neurons themselves initiate migraine attacks. Later studies by Leão, Olesen, Lauritzen, and others confirmed that neural dysfunction plays a primary role (AAP, 1944; Olesen, 1985; Lauritzen et al., 1983).

**FIGURE 2:**
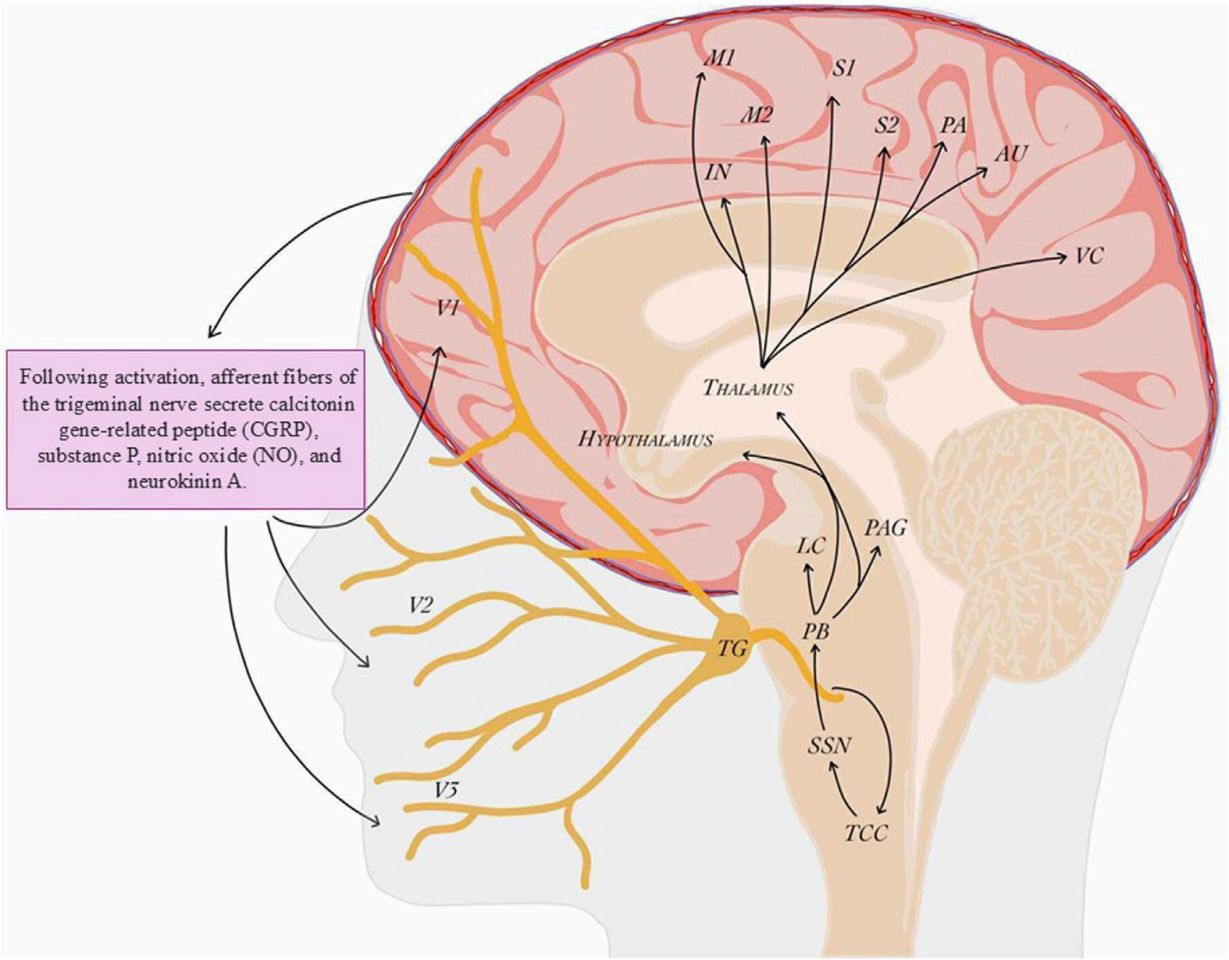
Trigeminovascular system and pathways of migraine pain transmission (created by BioRender; based on (Goadsby et al., 2017; Akerman et al., 2011)). Activation of the trigeminovascular system plays a central role in the pathophysiology of migraine. The headache phase begins with activation of meningeal nociceptive afferents: unmyelinated C fibers and thinly myelinated Aδ fibers arising mainly from the ophthalmic (V1) but also from the maxillary (V2) and mandibular (V3) divisions of the trigeminal nerve. These fibers densely innervate meningeal blood vessels and, upon activation, release vasoactive neuropeptides such as calcitonin gene-related peptide (CGRP), substance P, nitric oxide (NO), and neurokinin A. These mediators promote vasodilation, neurogenic inflammation, and peripheral sensitization. Nociceptive signals from trigeminal ganglion (TG) neurons are transmitted via the trigeminocervical complex (TCC) to higher brain centers, including the thalamus, hypothalamus, and cortical regions such as the somatosensory cortex (S1/S2), motor cortex (M1/M2), visual cortex (VC), insula (IN), parietal association cortex (PA), and auditory cortex (AU). Brainstem nuclei, including the locus coeruleus (LC), periaqueductal gray (PAG), parabrachial nucleus (PB), and superior salivatory nucleus (SSN), further modulate pain transmission, underscoring the complexity of the neural networks underlying migraine pathogenesis.

Modern research highlights the interplay between the trigeminovascular system (TVS) and central pain-processing networks (Figure 2). A pivotal breakthrough was the identification of the calcitonin gene–related peptide (CGRP) as a key mediator of migraine. Moskowitz’s theory of neurogenic inflammation and the work of Goadsby and Edvinsson in the early 1990s showed that CGRP drives meningeal vasodilation and pain transmission (Moskowitz, 1993; Goadsby and Edvinsson, 1994). Functional MRI studies performed during and between migraine attacks further revealed not only dynamic changes in intra- and extracranial arterial diameters but also sensory hypersensitivity in cortical regions—a hallmark unique to migraine (Schwedt et al., 2015; Messina et al., 2022).

The central nervous system is deeply involved in migraine. Brainstem structures, including the periaqueductal gray (PAG), locus coeruleus, and raphe nuclei, are hyperactive and modulate pain sensitivity. The hypothalamus, which regulates circadian rhythms and stress responses, shows early activation in the prodromal phase, explaining symptoms such as fatigue, mood changes, and food cravings before headache onset (Goadsby et al., 2017; Akerman et al., 2011; Bernstein and Burstein, 2012). Neuroimaging demonstrates that during attacks the hypothalamus couples strongly with the trigeminal nucleus, while between attacks its connections shift to other areas such as the PAG. These findings indicate that the hypothalamus, via reciprocal connections with the trigeminocervical complex, can trigger a migraine (Schulte et al., 2017).

Another hallmark of central involvement is cortical spreading depression (CSD) - a slowly propagating wave of neuronal depolarization, typically originating in the occipital lobe and advancing to parietal, temporal, and frontal regions (Bolay and Moskowitz, 2005). First described in 1944, CSD propagates across the cortex at a rate of 2–5 mm/min and is responsible for the aura experienced by many patients (Tfelt-Hansen, 2010; Lauritzen, 2010). EEG studies demonstrate a brief reduction in cortical activity lasting up to 60 s, followed by a period of transient hyperexcitability before normal function resumes. CSD can activate trigeminal nociceptors and contribute to prolonged central sensitization, a key factor in chronic migraine and in symptoms such as allodynia and hypersensitivity to light, sound, and smell (Zhang et al., 2010; Tang et al., 2020).

The headache phase begins with activation of meningeal nociceptive afferents -unmyelinated C fibers and thinly myelinated Aδ fibers originating from the ophthalmic (V1) division of the trigeminal nerve, which densely innervate the dura mater, arachnoid, and major cerebral arteries. Terminal endings of these fibers release vasoactive neuropeptides, notably CGRP, but also PACAP, substance P, nitric oxide (NO), neurokinin A, and vasoactive intestinal peptide (VIP). These mediators promote vasodilation and trigger neurogenic inflammation in adjacent meningeal and brain regions (Goadsby et al., 2017; Akerman et al., 2011; Zhang et al., 2010; Tang et al., 2020; Eftekhari et al., 2013; Schytz et al., 2009). Neurogenic inflammation amplifies the activity of trigeminal afferents, leading to peripheral and secondary central sensitization and producing the characteristic pulsating migraine pain. Trigeminal ganglion fibers relay signals to the spinal trigeminal nucleus, where second-order neurons project to the posterior thalamus. During attacks, the thalamus shows altered functional connectivity with cortical sensory areas. CGRP-driven neuromodulation and therapies that block CGRP or its receptor - can modulate thalamic activity and reduce the sensory hypersensitivity underlying photophobia and other migraine symptoms (Schulte and May, 2017; Tu et al., 2016; Ziegeler et al., 2020).

Recognition of CGRP’s central role revolutionized migraine treatment. Anti-CGRP therapies, including monoclonal antibodies and the newer second- and third-generation gepants, were developed specifically for migraine. These agents, which target either CGRP or its receptor, have shown high efficacy and a favorable safety profile for both acute treatment and long-term prevention of episodic and chronic migraine (Edvinsson and Warfvinge, 2019).

## 3 Understanding the CGRP pathway in the migraine

**FIGURE 3:**
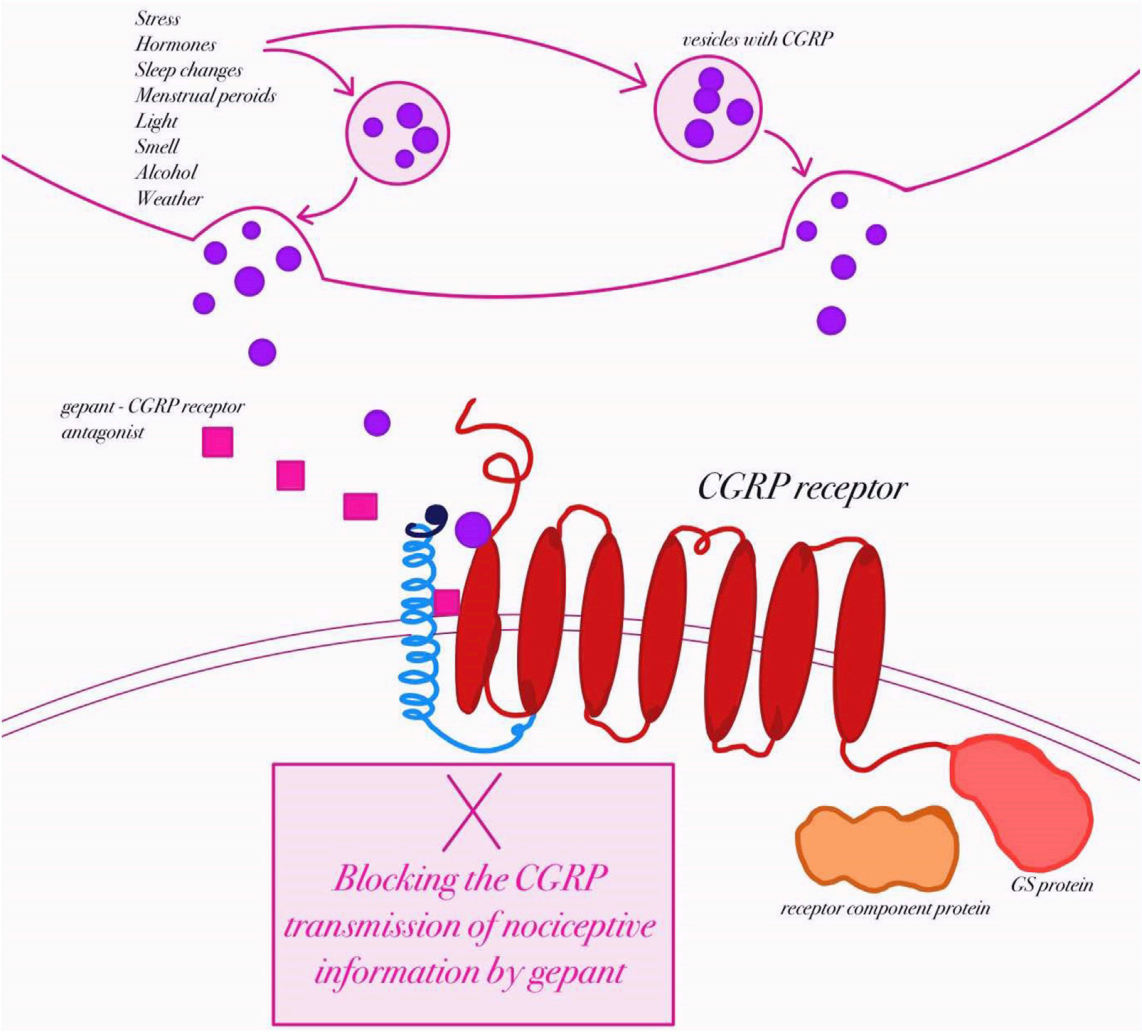
Mechanism of action of gepants in migraine therapy (created by BioRender, based on (Edvinsson, 2017)). Schematic representation of calcitonin gene–related peptide (CGRP) release from trigeminal nerve terminals in response to potential migraine triggers (e.g., stress, hormonal changes, light, smell, alcohol, weather). Upon release, CGRP binds to its receptor, a heterotrimeric complex consisting of the calcitonin receptor-like receptor (CLR), receptor activity–modifying protein 1 (RAMP1), and receptor component protein (RCP), which activates intracellular Gs-protein–mediated signaling. Gepants are small-molecule antagonists that block CGRP binding at its receptor, thereby preventing the transmission of nociceptive information and attenuating migraine pathophysiology.

Calcitonin gene-related peptide (CGRP) is a 37–amino acid peptide derived from the calcitonin gene, and it exists in two isoforms: α-CGRP, which is primarily found in the central and peripheral nervous systems, and β-CGRP, which is more commonly expressed in the enteric nervous system (Messlinger et al., 1993; Amara et al., 1982). CGRP is widely distributed throughout the sensory nervous system, particularly in the trigeminal ganglion (Eftekhari et al., 2015). CGRP is expressed in both the peripheral and central nervous systems and is released by nerve fibers running along meningeal and cerebral arteries as well as other blood vessels (Messlinger et al., 1993; Edvinsson, 2021). Its discovery in the trigeminovascular system in 1985 first linked CGRP to disturbances in neuronal homeostasis associated with migraine (McCulloch et al., 1986; Edvinsson et al., 1987). In 1990, Goadsby and colleagues demonstrated elevated levels of CGRP in the extracerebral circulation during migraine attacks, providing early evidence of its key role in migraine pathophysiology (Goadsby et al., 1990). Research has shown that CGRP is expressed in more than half of the trigeminal fibers within the central nervous system and in approximately 35%–50% of neurons in the trigeminal ganglion (Eftekhari et al., 2010; Lennerz et al., 2008). Receptors for CGRP have also been identified in the ganglion’s satellite cells (Thalakoti et al., 2007). CGRP exists in two highly similar isoforms: α-CGRP and β-CGRP, whose biological actions are nearly indistinguishable (Amara et al., 1982; Russell et al., 2014). They are encoded by the CALCA and CALCB genes, respectively; however, regulation of CALCB expression, responsible for β-CGRP synthesis (primarily in the enteric nervous system), remains unclear (Hay et al., 2018; Russo and Hay, 2023). The α-CGRP isoform predominates in the trigeminal ganglion and is a mature 37–amino acid peptide characterized by a disulfide bridge and an amidated carboxyl terminus (Amara et al., 1982). These structural motifs, shared across the CGRP peptide family, are essential for receptor activation. The broader CGRP gene family also encodes related peptides such as amylin, adrenomedullin (AM), and adrenomedullin-2 (AM2, also known as intermedin), each with distinct functions and tissue distribution (Mulderry et al., 1988). Beyond the nervous system, CGRP is present in the cardiovascular and gastrointestinal systems, where it exerts diverse physiological effects (Franco-Cereceda et al., 1987). CGRP receptors are G-protein–coupled receptors (GPCRs) composed of a seven-transmembrane calcitonin receptor-like receptor (CLR) paired with a single transmembrane receptor activity-modifying protein (RAMP) (Wattiez et al., 2020) (Figure 3). CLR combined with RAMP1 forms the canonical CGRP receptor, while combinations with RAMP2 or RAMP3 create the adrenomedullin receptors ADM1 and ADM2, respectively (McLatchie et al., 1998; Hay et al., 2006). Although all members of this receptor family signal through G proteins, their differing molecular architectures may produce distinct pharmacological responses to receptor-targeted therapies (Hay et al., 2006). Importantly, calcitonin receptor (CTR) can also combine with RAMP1 to form the amylin 1 (AMY_1_) receptor. This receptor is classically considered an amylin receptor but CGRP is equipotent to amylin in activating the AMY_1_ receptor (Olesen et al., 2009; Brain and Grant, 2004; Bhakta et al., 2021; Labastida-Ramírez et al., 2023). In fact, CGRP binds with high affinity to CTR/RAMP1 (AMY_1_) in addition to CLR/RAMP1, which has important implications: some gepants designed to block CGRP signaling may cross-react with AMY_1_ receptor complexes (Olesen et al., 2009; Knight and Goadsby, 2001; Thomsen et al., 1994). Because CTR/RAMP1 and CLR/RAMP1 share the same RAMP1 subunit, pharmacological agents (especially gepants) sometimes lack perfect selectivity and may antagonize both canonical CGRP receptors and AMY_1_ receptor complexes (Olesen et al., 2009; Brain and Grant, 2004).

During a migraine attack, CGRP is released from activated trigeminal sensory fibers that innervate the meninges and cerebral blood vessels (Olesen et al., 2009). This release causes marked vasodilation of intracranial arteries and promotes neurogenic inflammation through mast-cell activation and the release of inflammatory mediators (Brain and Grant, 2004). CGRP also enhances excitability of second-order neurons in the trigeminocervical complex and the thalamus, amplifying pain transmission to higher cortical centers (Goadsby and Edvinsson, 1994; Knight and Goadsby, 2001). Intravenous infusion of CGRP induces delayed migraine attacks in susceptible individuals, but not in healthy controls, indicating that CGRP amplifies downstream signaling cascades rather than initiating the migraine itself (Thomsen et al., 1994; Amin et al., 2013). Multiple studies have reported elevated CGRP concentrations in the jugular vein during migraine attacks (Goadsby et al., 1990; Edvinsson and Goadsby, 2019), and higher interictal levels have been detected in the blood, saliva, and tear fluid of migraine patients compared with controls (Cernuda-Morollón et al., 2013; Gallai et al., 1995; Kaiser et al., 2012). Injection of CGRP into the posterior thalamus activates neurons and triggers pain-signaling cascades (Noseda and Burstein, 2013). Likewise, in mice engineered to express the human RAMP1 subunit of the CGRP receptor, peripheral or central administration of CGRP provokes photophobia and drives the animals to seek darkness, behavior reminiscent of human migraine attacks (Recober et al., 2009).

These findings highlight CGRP as a pivotal mediator of trigeminovascular activation and migraine pain, though the precise mechanisms by which it precipitates attacks remain incompletely understood (Edvinsson et al., 2022). In the meninges, CGRP likely promotes neurogenic inflammation by sensitizing neurons and activating mast cells, resulting in meningeal vasodilation and a self-perpetuating loop of nociceptor sensitization (Olesen et al., 2009; Brain and Grant, 2004). Modulating neuronal activity in this environment can disrupt this positive feedback cycle.

Therapeutically, antagonists that block CGRP or its receptor have shown high efficacy in more than half of migraine patients (Dodick et al., 2019). By preventing CGRP from binding to its receptor, these agents reduce the release of vasoactive substances and nociceptive transmitters, thereby lowering both the frequency and intensity of migraine attacks (Goadsby et al., 2020). While much of the mechanistic insight comes from preclinical studies, the accumulating clinical evidence underscores the importance of continued research into CGRP’s receptor interactions and signaling pathways, with the goal of improving and personalizing migraine treatment (Edvinsson, 2021).

## 4 Gepants: a new era in migraine therapy

For decades, the pharmacological management of migraine relied primarily on non-specific analgesics such as non-steroidal anti-inflammatory drugs (NSAIDs) and the serotonin 5-HT1B/1D receptor agonists known as triptans (Tfelt-Han et al., 2000; Tfelt-Hansen, 2010). While effective for many patients, these therapies are limited by incomplete efficacy (Goadsby et al., 2017) and, in the case of triptans, by vasoconstrictive effects that restrict their use in individuals with cardiovascular disease (Dodick et al., 2010).

The discovery in the late 1990s of calcitonin gene–related peptide (CGRP) as a key mediator of migraine attacks opened the door to a new, targeted therapeutic strategy. In 2004, Boehringer Ingelheim reported the first clinical data on CGRP receptor antagonists, introducing the small-molecule class now known as gepants (Olesen et al., 2004). Gepants are small-molecule (<1 kDa) antagonists of the CGRP receptor that selectively inhibit receptor activation within the trigeminal ganglion, trigeminal fibers, and dura mater (Ailani et al., 2021a). By preventing CGRP from binding to its receptor, they interrupt synaptic pain transmission at an early stage of the migraine cascade. Their receptor-binding affinities range from approximately 0.1 nM to 0.8 nM, reflecting potent and specific inhibition (Moore and Salvatore, 2012). Unlike triptans, gepants do not induce constriction of cerebral or coronary arteries. Beyond their lack of vasoconstrictive activity, evidence suggests that gepants may provide cardiovascular benefits: by blocking the calcitonin receptor–like receptor (CLR), they reduce the release of vasodilatory substances, potentially lowering vascular stress and the risk of cerebral ischemia or heart failure (Smillie et al., 2014; Schebesch et al., 2013; Li et al., 2013; Mulder et al., 2020; Deen et al., 2017). Due to the absence of vasoconstrictive effects, they are drugs with a lower cardiovascular risk than triptans (contraindicated in patients with, among others, coronary artery disease), however, it should be noted that blocking CGRP or its receptor with gepants/CGRP monoclonal antibodies may worsen ischaemia and related conditions such as hypertension. (Verheggen et al., 2002; Chan et al., 2010).

Three pharmacological generations of gepants have been developed, each improving upon the last in efficacy and safety. The first generation was limited by significant hepatotoxicity and poor oral bioavailability. Olcegepant (BIBN-4096), the earliest compound investigated, required intravenous administration, while other first-generation agents such as BI 44370 TA, MK-3207, and telcagepant (MK-0974) demonstrated inadequate oral absorption and were discontinued by 2010 due to limited clinical effectiveness and hepatic concerns. These challenges stimulated the creation of second-generation gepants—including atogepant, ubrogepant, and rimegepant—which achieved higher efficacy and markedly improved safety, with no significant evidence of hepatotoxicity. Among these, rimegepant is uniquely approved by the U.S. Food and Drug Administration (FDA) for both acute and preventive migraine treatment (Olesen et al., 2004; Ho et al., 2008; Scott, 2020a; Woodhead et al., 2022).

**FIGURE 4:**
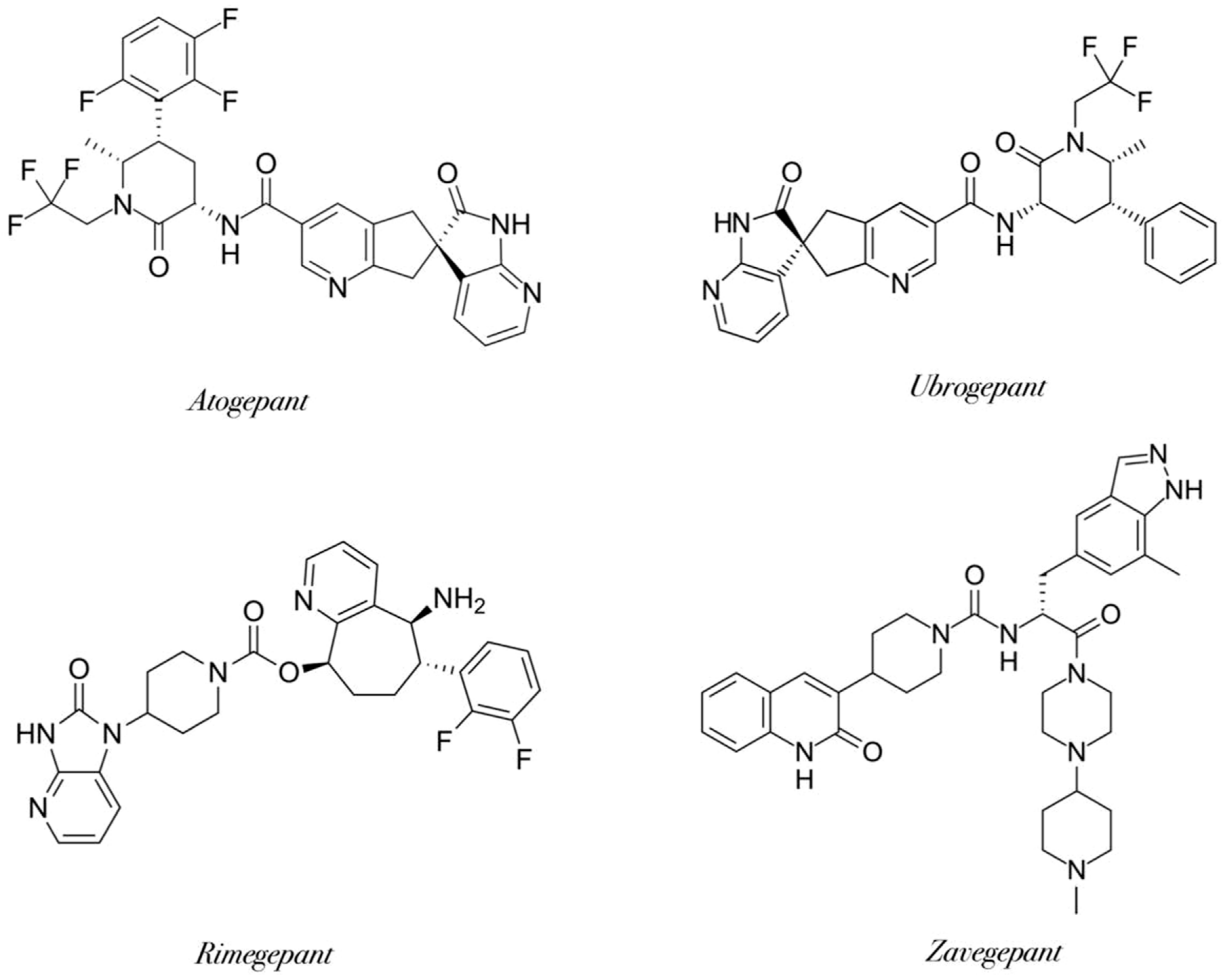
Chemical structures of clinically approved gepants (created by BioRender; based on (IUPHAR/BPS Guide to Pharmacology, 2025a; IUPHAR/BPS Guide to Pharmacology, 2025b; IUPHAR/BPS Guid e to Pharmacology, 2025c; IUPHAR/BPS Guide to Pharmacology, 2025d)). Representative small-molecule CGRP receptor antagonists currently approved for clinical use include atogepant, ubrogepant, rimegepant, and zavegepant. Despite structural diversity, these molecules share the ability to selectively block the CGRP receptor, thereby preventing receptor activation and downstream signaling in the trigeminovascular system. Structural modifications between successive generations have improved oral bioavailability, metabolic stability, and safety, reducing hepatotoxicity observed with earlier compounds.

More recently, zavegepant has emerged as the first intranasal third-generation gepant to receive FDA approval, offering rapid onset of action and alternative delivery for patients who cannot tolerate oral therapy. Today, gepants are available as oral tablets, orally disintegrating tablets, and intranasal sprays, providing versatile options for individualized migraine management and firmly establishing this class as a cornerstone of modern migraine pharmacotherapy. Representative small-molecule CGRP receptor antagonists currently approved for clinical use include atogepant, ubrogepant, rimegepant, and zavegepant (Figure 4). Despite structural diversity, these molecules share the ability to selectively block the CGRP receptor, thereby preventing receptor activation and downstream signaling in the trigeminovascular system. Structural modifications between successive generations have improved oral bioavailability, metabolic stability, and safety, reducing hepatotoxicity observed with earlier compounds.

### 4.1 Atogepant (AGN-241689, MK-8031)

Atogepant, a second-generation gepant structurally distinct from earlier agents such as telcagepant, is an orally administered small-molecule antagonist of the calcitonin gene-related peptide (CGRP) receptor. Developed by AbbVie, it is indicated for the preventive treatment of migraine. In September 2021, the FDA approved atogepant for episodic migraine prevention in adults, and in April 2023 the approval was extended to chronic migraine (Deeks, 2022). It is marketed as Qulipta in oral doses of 10, 30, and 60 mg.

Clinical development of atogepant began in 2019, and to date more than a dozen trials have assessed its safety and efficacy. In the pivotal phase 2b/3 trial NCT02848326 (1,772 participants), patients received placebo or atogepant (10–60 mg once or twice daily) for 12 weeks (Goadsby et al., 2020; ClinicalTrials.gov, 2025a; Rizzoli et al., 2024). All active treatment groups achieved greater reductions in monthly migraine days (MMDs) compared with placebo (e.g., −4.0 days with 10 mg QD vs. −2.9 with placebo). The most common adverse events were nausea (5%–12%) and fatigue (1%–10%) (Goadsby et al., 2020). The large phase 3 study NCT03777059 (2,270 patients with 4–14 migraine days/month) confirmed these results (Lipton et al., 2023c). Once-daily atogepant (10, 30, or 60 mg) produced dose-dependent reductions in MMDs over 12 weeks (−3.7 to −4.2 vs. −2.5 with placebo). At the higher doses, significant improvements were also observed in quality of life (AIM-D scores) (Gottschalk et al., 2024). The most frequent adverse effects were nausea and constipation (Rizzoli et al., 2024; Ailani et al., 2021b; Schwedt et al., 2022; Lipton et al., 2024). In chronic migraine, the phase 3 trial NCT03855137 (778 patients across 142 centers) demonstrated meaningful efficacy. At 12 weeks, atogepant 30 mg BID and 60 mg QD reduced MMDs by −7.5 and −6.9 respectively, compared with −5.1 with placebo. Least squares mean differences favored both dosing regimens, and treatment was well tolerated (Rizzoli et al., 2024; Clin icalTrials.gov, 2025c). Long-term efficacy was confirmed in the one-year trial NCT03700320, where daily atogepant 60 mg reduced MMDs from −3.8 days in weeks 1–4 to −5.2 in weeks 49–52. Improvements were also reflected in patient-reported outcomes, including MSQ v2.1 and AIM-D. The most frequent adverse events were mild to moderate, such as upper respiratory tract infection, constipation, nausea, and urinary tract infection (Rizzoli et al., 2024; Lipton et al., 2024; ClinicalTrials.gov, 2025b). An additional publication of NCT03777059 focused on patient-reported outcomes, particularly MSQ v2.1 (role function-restrictive domain, RFR) and AIM-D (physical impairment [PI] and performance of daily activities [PDA]) (Rizzoli et al., 2024; Ailani et al., 2021b; Schwedt et al., 2022; Lipton et al., 2024). In this randomized, double-blind, multicenter study involving 910 participants, atogepant groups included 214 (10 mg), 223 (30 mg), and 222 (60 mg) patients. All atogepant groups showed significantly greater improvements vs. placebo in MSQ-RFR at week 12, with dose-response relationships favoring higher doses. AIM-D PI and PDA improvements were significant in the 30 mg group. Exploratory analyses indicated that atogepant 30 mg and 60 mg produced clinically meaningful benefits in MSQ-RFR and AIM-D outcomes, while the 10 mg dose showed modest improvements compared with placebo. The earliest nominal improvements in MSQ domains, AIM-D, and HIT-6 were observed at week 4 with the 30 mg and 60 mg doses (Rizzoli et al., 2024; Ailani et al., 2021b; Schwedt et al., 2022; Lipton et al., 2024) (see also Table 1).

**TABLE 1:** Recent clinical trials of second- (atogepant, ubrogepant, rimegepant) and third-generation (zavegepant) gepants in migraine therapy: atogepant and ubrogepant.

Trial ID	Study type	Objective/Methods	Participants (N) and criteria	Treatment arms	Adverse events (TEAEs)	Duration	Main findings	Reference
Atogepant								
NCT02848326	Phase 2b/3, randomized, double-blind, multicenter	Efficacy/safety in episodic migraine prevention	N = 1772; 4–14 migraine days/month	Placebo vs. Atogepant 10–60 mg QD or BID	Nausea (5%–12%), fatigue (1%–10%)	12 weeks	All doses significantly reduced MMDs vs. placebo (e.g., −4.0 vs. −2.9 days)	Dodick et al. (2019); IUPHAR/BPS Guide to Pharmacology (2025d)
NCT03777059	Phase 3, randomized, double-blind, multicenter	Effect on patient-reported outcomes (MSQ v2.1, AIM-D, HIT-6) and reduction in monthly migraine days (MMDs) vs. placebo	N = 873; adults (18–80 years), ≥1-year history of migraine (with/without aura), 4–14 migraine days/month	Atogepant 10 mg (n = 214), 30 mg (n = 223), 60 mg (n = 222); Placebo (n = 214), QD	Constipation: 6.9%–7.7%; Nausea: 4.4%–6.1%	12 weeks	Atogepant significantly reduced MMDs vs. placebo (−3.7 to −4.2 vs. −2.5 days). Improvements observed in MSQ-RFR and AIM-D domains	Chan et al. (2010); Deeks (2022); ClinicalTrials.gov (2025a); Rizzoli et al. (2024)
NCT03700320	Phase 3, open-label, multicenter	Long-term efficacy and safety, ≥50%, ≥75%, 100% reduction in MMDs	N = 744; adults (18–80 years), 4–14 migraine days/month	Atogepant 60 mg (n = 546); Standard therapy (n = 198), QD	Respiratory tract infection (10.3%), constipation (7.2%), nausea (6.3%), UTI (5.2%); Serious TEAEs 4.4%	52 weeks	Sustained reduction in MMDs up to week 52; ≥50% reduction achieved in 84% at week 52. Significant QoL improvements	Goadsby et al. (2020); ClinicalTrials.gov (2025a); Rizzoli et al. (2024)
NCT03855137	Phase 3, randomized, double-blind, placebo-controlled	Efficacy/safety in chronic migraine	N = 755; adults (18–80 years), ≥15 headache days/month, ≥1-year migraine history	Atogepant 30 mg BID (n = 257), 60 mg QD (n = 262); Placebo (n = 259)	Constipation: 10%–11%; Nausea: 8%–10%; Weight loss ≥7%: ∼6%	12 weeks	Atogepant reduced MMDs (−7.5 and −6.9) vs. placebo (−5.1). Significant benefit across both dosing regimens	Ho et al. (2008); Lipton et al. (2023c)
Ubrogepant								
NCT01657370	Phase 2b, randomized, double-blind, multicenter	Safety and efficacy in acute migraine	N = 516; adults with migraine	Ubrogepant 100 mg; Placebo, single dose during a migraine attack (no regular dosing)	Mostly mild TEAEs; no hepatotoxicity	Single attack	Ubrogepant superior to placebo for pain relief and pain freedom; no liver signal	Gottschalk et al. (2024); American Headache Society (2021)
NCT01613248	Phase 2, randomized	Dose-ranging efficacy	N = 834; adults with migraine	Ubrogepant 1–100 mg; Placebo, single dose during a migraine attack (no regular dosing)	Nausea, dry mouth, fatigue, somnolence	Single attack	25–100 mg effective; 2 h pain freedom 25.5% vs. 8.9% placebo	AbbVie (2023b); Ailani et al. (2021b)
NCT02867709 (ACHIEVE II)	Phase 3, randomized, double-blind, placebo-controlled	Acute treatment efficacy, pain freedom and MBS at 2 h	N = 1465; adults (18–75 years), ≥1-year migraine history	Ubrogepant 25 mg (n = 561), 50 mg (n = 562); Placebo (n = 563), single dose during a migraine attack (no regular dosing)	Mild TEAEs; no hepatic signal	Single attack	Pain freedom at 2 h: 20.7%–21.8% vs. 14.3% with placebo. Significant benefit in MBS freedom	Rizzoli et al. (2024); Schwedt et al. (2022)
NCT02873221	Phase 3, repeat-dose	Efficacy across multiple attacks (up to 8)	N = 808; 19,291 attacks	Ubrogepant 50/100 mg; Placebo, single dose during a migraine attack (no regular dosing)	Rare serious TEAEs (2%–3%)	Multiple attacks	Greater efficacy when taken during mild pain (2 h pain freedom: 47.1% with 50 mg; 55.2% with 100 mg)	Lipton et al. (2024); ClinicalTrials.gov (2025b)
NCT04492020	Phase 3, randomized	Effect in prodromal phase	N = 518; adults experienced 2–8 migraine attacks per month (with moderate-to-severe headache among the attacks)	Ubrogepant 100 mg; Placebo	Mild TEAEs comparable to placebo	Single prodromal dose	Reduced progression to moderate/severe headache: 46% vs. 29% placebo	ClinicalTrials.gov (2025c); ClinicalTrials.gov (2025d)
NCT04818515	Phase 1b, open-label, fixed-sequence study	PK/safety of coadministration with atogepant	N = 26; ≥15 up to 62 years, history of migraine ≥1 year, at least 2 migraine attacks per month for the 2 months prior to screening	Ubrogepant + Atogepant (oral tablets of ubrogepant followed by oral tablets of atogepant)	No relevant interactions	Short-term	Safe; no pharmacokinetic interactions observed	Li et al. (2020)
Rimegepant								
NCT03461757	Phase 3, randomized, double-blind placebo-controlled	Acute treatment efficacy of rimegepant 75 mg ODT	N = 1811; adults ≥18 years, ≥1-year migraine history	Rimegepant 75 mg (n = 732); Placebo (n = 734), single dose during a migraine attack (no regular dosing)	Nausea (2% vs. <1%); UTI (1% vs. 1%)	Single attack	Pain freedom at 2 h: 21% vs. 11% with placebo. Significant functional improvement	ClinicalTrials.gov (2025e); Voss et al. (2016)
NCT03237845	Phase 3, randomized, double-blind, placebo-controlled, multicenter	Replication of acute efficacy study	N = 1499; adults with migraine (with or without aura), migraine history of ≥1 year, 2–8 migraine attacks per month during the baseline period	Rimegepant 75 mg ODT; Placebo, single dose during a migraine attack (no regular dosing)	Similar safety to placebo	Single attack	Confirmed efficacy; freedom from MBS at 2 h	ClinicalTrials.gov (2025f); Lipton et al. (2019)
NCT03732638	Phase 2/3, randomized, double-blind, placebo-controlled, multicenter	Preventive treatment efficacy	N = 1591; adults, ≥1-year migraine history	Rimegepant 75 mg EOD (n = 373); Placebo (n = 374), single dose during a migraine attack (no regular dosing)	Nasopharyngitis (4% vs. 2%); nausea (3% vs. 1%)	12 weeks	Reduction in MMDs (−4.3 vs. −3.5 with placebo). Statistically significant benefit	ClinicalTrials.gov (2025g); Lipton et al. (2022)
Zavegepant								
NCT04571060	Randomized, double-blind, placebo-controlled	Acute treatment efficacy, safety of intranasal formulation	N = 1978; adults, ≥1-year migraine history	Zavegepant 10 mg intranasal (n = 703); Placebo (n = 702), single dose during a migraine attack (no regular dosing)	Dysgeusia (21% vs. 5%); nasal discomfort (4% vs. 1%); nausea (3% vs. 1%)	Single attack	Pain freedom at 2 h: 24% vs. 15% with placebo. Rapid onset of action, favorable tolerability	ClinicalTrials.gov (2025h); Dodick et al. (2023)
NCT04408794	Phase 2/3, randomized, open-label, multicenter	Safety and tolerability of repeated intranasal use	N = 603; adult patients diagnosed with migraine (with or without aura)	Zavegepant intranasal 10 mg, 20 mg, administered QD for up to 14 days or as two sequential doses for up to 8 days	Dysgeusia, nasal discomfort, nausea	Long-term	Well tolerated; no hepatotoxicity observed	ClinicalTrials.gov (2025i); Center for Drug Evaluation and Research and FDA (2024)

Abbreviations: QD (quaque die) - once daily; BID (bis in die) - twice daily; MMDs, monthly migraine days; MSQ, v2.1 - Migraine-Specific Quality of Life Questionnaire, version 2.1; MSQ-RFR, Migraine-Specific Quality of Life Questionnaire–Role Function-Restrictive domain; AIM-D, Activity Impairment in Migraine–Diary; HIT-6, Headache Impact Test – 6 items; UTI, Urinary Tract Infection; MBS, Most Bothersome Symptom; TEAEs, Treatment-Emergent Adverse Events; QoL - Quality of Life; ODT, Orally Disintegrating Tablet.

Across studies, atogepant consistently reduced migraine frequency, improved quality of life, and demonstrated a favorable safety profile. Benefits were dose-dependent, with the 30 mg and 60 mg regimens showing the strongest and most sustained efficacy in both episodic and chronic migraine. In September 2021, the FDA approved atogepant for the preventive treatment of episodic migraine. In April 2023, the approval was extended to include chronic migraine prevention. Atogepant is marketed under the brand name Qulipta, available in oral doses of 10, 30, and 60 mg (American Headache Society, 2021; AbbVie, 2023b).

### 4.2 Ubrogepant (MK-1602)

Ubrogepant is a novel gepant developed for the acute treatment of migraine. To date, 12 clinical trials have evaluated its efficacy and safety. Compared with atogepant, it differs in chemical structure, time to maximum plasma concentration (∼1.5–2 h vs. ∼1–2 h), and half-life (5–7 h vs. ∼11 h), leading to minimal or no accumulation with repeated dosing.

The first study (MK-1602-005) was a phase 1 trial in postmenopausal women, followed by population pharmacokinetic and exposure–response studies. Safety evaluation was a major focus, particularly regarding hepatotoxicity. In the placebo-controlled, double-blind, multicenter trial NCT01657370 (516 participants), patients received placebo or ubrogepant 100 mg. Most treatment-emergent adverse events (TEAEs) were mild (89% in both groups); moderate TEAEs occurred in 11% (placebo) vs. 8% (ubrogepant). Reported events included oropharyngeal pain. No signal of liver injury was observed (ClinicalTrials.gov, 2025d; Li et al., 2020). Efficacy was confirmed in NCT01613248, a randomized trial with 834 participants assigned to placebo or ubrogepant (1–100 mg). The most frequent TEAEs were nausea, dry mouth, fatigue, and somnolence, occurring at rates comparable to triptans but without chest or throat discomfort. Two serious TEAEs (hypertension, myoclonus) occurred at 50 mg. Ubrogepant at 25–100 mg demonstrated superiority over placebo, with statistically significant 2-h pain freedom (25.5% vs. 8.9%; p = 0.003) and sustained pain relief with 25 mg and 50 mg (ClinicalTrials.gov, 2025e; Voss et al., 2016). Larger phase 3 trials further validated its efficacy. In NCT02867709 (1,465 participants), 2-h pain freedom rates were 11.8% with placebo, 19.2% with 50 mg (p = 0.002), and 21.2% with 100 mg (p < 0.001). Pain relief at 2 h occurred in 60%–61% with ubrogepant vs. 49% with placebo. A related study using 25 mg and 50 mg confirmed dose-dependent superiority over placebo (ClinicalTrials.gov, 2025f; Lipton et al., 2019). In NCT02873221, 808 participants treated up to eight attacks (19,291 in total). Efficacy was higher when medication was taken during mild pain (2-h pain freedom: 47.1% with 50 mg; 55.2% with 100 mg) compared with moderate pain (23.6% and 26.1%, respectively). Photophobia, phonophobia, and nausea resolution were also more frequent when treating early attacks. Serious TEAEs were rare (2%–3%) (ClinicalTrials.gov, 2025g; Lipton et al., 2022). The prodromal-phase trial NCT04492020 showed that ubrogepant 100 mg reduced progression to moderate/severe headache within 24 h in 46% of prodromal events vs. 29% with placebo. Adverse events were mild and comparable between groups (ClinicalTrials.gov, 2025h; Dodick et al., 2023). A phase 1b study (NCT04818515) demonstrated that coadministration of ubrogepant with atogepant had no clinically meaningful pharmacokinetic interaction and was well tolerated (ClinicalTrials.gov, 2025i) (see also Table 1).

Ubrogepant should not be used during pregnancy or in end-stage renal disease. It is not indicated for migraine prevention (Center for Drug Evaluation and Research and FDA, 2024; Dhir, 2020). Concomitant use with strong CYP3A4 inhibitors (e.g., clarithromycin, erythromycin, itraconazole, ketoconazole, diltiazem, grapefruit) increases ubrogepant exposure, while CYP3A4 inducers (e.g., rifampin, phenytoin, phenobarbital, St. John’s wort, glucocorticoids) reduce it (Medscape, 2020; Scott, 2020b).

Overall, ubrogepant has demonstrated a favorable safety and efficacy profile, particularly when administered early in the course of a migraine attack (Center for Drug Evaluation and Research and FDA, 2024). In December 2019, it was approved in the United States for acute migraine treatment and is marketed by Allergan (AbbVie) under the brand name Ubrelvy, available in 50 mg and 100 mg oral tablets (AbbVie, 2023a).

### 4.3 Rimegepant (BMS-927711; BHV-3000)

Rimegepant is a second-generation, orally disintegrating small-molecule CGRP receptor antagonist (Croop et al., 2021a). To date, 14 clinical trials have been conducted. It was approved by the FDA in July 2019 and is the only gepant authorized in Europe for both the acute treatment of migraine (with or without aura) and preventive therapy for episodic migraine in adults, making it unique among gepants with proven efficacy in both indications (ClinicalTrials.gov, 2025j; Lipton et al., 2023d).

Rimegepant binds with high affinity to the human CGRP receptor, and *in vitro* also interacts with the AMY1 receptor (ClinicalTrials.gov, 2025k; Mullin et al., 2024). While its precise therapeutic mechanism remains incompletely understood, clinical efficacy has been demonstrated in multiple trials. In two pivotal phase 3 studies (NCT03461757, NCT03237845) including over 2,400 patients, a single 75 mg dose of rimegepant was superior to placebo for 2-h pain freedom (21% vs. 11%) and return to normal function (38.1% vs. 25.8%). The most common adverse events (AEs) were nausea and urinary tract infection (ClinicalTrials.gov, 2025i; Center for Drug Evaluation and Research and FDA, 2024; Dhir, 2020). Preventive efficacy has been demonstrated in the phase 2/3 trial NCT03732638 (n = 695), where rimegepant 75 mg every other day significantly reduced monthly migraine days (−4.3 vs. −3.5 with placebo, weeks 9–12) (see also Table 1).

Based on these results, rimegepant (Nurtec ODT 75 mg, Biohaven Pharmaceuticals) was approved by the FDA in February 2020 for acute treatment, and in May 2021 its label was expanded to include preventive therapy for episodic migraine (Croop et al., 2019a; Clinic alTrials.gov, 2025l; Croop et al., 2019b; ClinicalTrials.gov, 2025m).

As rimegepant is primarily metabolized via CYP3A4, coadministration with strong CYP3A4 inhibitors (e.g., ketoconazole, clarithromycin) may increase its plasma levels, while strong CYP3A4 inducers (e.g., rifampin, St. John’s wort) may reduce its efficacy. Dose adjustment or caution may be required in such cases (Croop et al., 2021b).

### 4.4 Zavegepant (BHV-3500, BMS-742413)

Zavegepant is the first third-generation gepant and the only calcitonin gene–related peptide (CGRP) receptor antagonist formulated for intranasal administration. In March 2023, the U.S. Food and Drug Administration (FDA) approved zavegepant nasal spray (ZAVZPRET) for the acute treatment of migraine with or without aura in adults. Its intranasal delivery provides a rapid route of absorption, addressing a clinical need for patients experiencing nausea, vomiting, or delayed gastric emptying during migraine attacks (Croop et al., 2021a; ClinicalTrials.gov, 2025n). The pivotal phase 3 randomized, double-blind, placebo-controlled trial (NCT04571060) enrolled 1,269 participants with a history of migraine. Patients were randomized to receive a single 10 mg intranasal dose of zavegepant or placebo. At 2 h post-dose, 24% of patients in the zavegepant group achieved pain freedom compared with 15% in the placebo group (147 vs. 96 participants), a statistically significant difference that met the co-primary endpoint. Secondary outcomes included freedom from the most bothersome symptom at 2 h and sustained relief up to 48 h. Post-hoc analyses further demonstrated early onset of benefit, with separation from placebo as early as 15 min in some participants, underscoring the advantage of intranasal delivery for rapid migraine relief (ClinicalTrials.gov, 2025j; Lipton et al., 2023d). Additional clinical investigations have expanded the evidence base for zavegepant. A long-term safety and tolerability study (NCT04408794) evaluated repeated intranasal dosing, confirming favorable tolerability without hepatotoxicity, a concern associated with earlier gepant molecules. Dysgeusia, nasal discomfort, and nausea were the most commonly reported adverse events, while cardiovascular safety was preserved due to the absence of vasoconstrictive properties. Pharmacokinetic studies and phase 2/3 trials consistently supported its efficacy and safety profile, culminating in regulatory approval. Beyond migraine, exploratory studies have examined potential applications of zavegepant in other disease settings, although its clinical development remains centered on acute migraine therapy (ClinicalTrials.gov, 2025k; Mullin et al., 2024) (see also Table 1).

Taken together, zavegepant represents an important addition to the therapeutic armamentarium for acute migraine. Its intranasal formulation provides a non-oral, rapid-onset option suitable for patients with contraindications to triptans or those unable to tolerate oral medications during attacks. The robust efficacy and favorable safety profile demonstrated in phase 3 and long-term studies highlight zavegepant’s role as the most recent advancement in the gepant class and the first intranasal CGRP receptor antagonist available in clinical practice.

## 5 Concluding remarks

The development of oral second-generation gepants and the intranasal third-generation gepant has markedly expanded therapeutic options for migraine management. These agents provide effective alternatives for patients who do not achieve satisfactory relief with triptans or for those at increased cardiovascular risk, as gepants do not induce vasoconstriction and are therefore safer in this regard. Importantly, current studies have not demonstrated a correlation between second-generation gepants and hepatotoxicity. The most commonly reported adverse events include constipation, nausea, and occasional infections of the urinary or respiratory tract. More serious but infrequent adverse events such as hypertension, optic neuritis, weight loss, and asthma have been described. While existing trials confirm the efficacy and favorable safety profile of gepants, further research is needed to assess the long-term safety of these agents and their potential role relative to triptans, as no direct head-to-head trials have yet been performer (Table 2).

**TABLE 2:** Comparison of gepants and triptans in migraine management (based on (Dodick et al., 2019; Goadsby et al., 2020; Ailani et al., 2021b; Lipton et al., 2019; Scott, 2020b; AbbVie, 2023a; Croop et al., 2021a; ClinicalTrials.gov, 2025j; Lipton et al., 2023d; ClinicalTrials.gov, 2025k; Mullin et al., 2024)).

Feature	Gepants (e.g., ubrogepant, rimegepant, atogepant)	Triptans (e.g., sumatriptan, rizatriptan, zolmitriptan)
Mechanism of action	CGRP receptor antagonists → block calcitonin gene–related peptide signaling (involved in migraine pathophysiology)	5-HT1B/1D receptor agonists → vasoconstriction and inhibition of neuropeptide release
Onset of action	Generally slower than triptans (may take 1–2 h)	Faster onset (30–60 min for oral, faster with intranasal or subcutaneous forms)
Efficacy (acute treatment)	Effective for pain relief and associated symptoms; less robust than triptans in some head-to-head studies	Highly effective for acute migraine; long history of use
Use in prevention	Some gepants (e.g., atogepant, rimegepant) approved for preventive therapy	Approved for menstrual related migraine (mini prophylaxis)
Cardiovascular safety	No vasoconstriction → safe in patients with cardiovascular disease	Contraindicated in patients with CAD, stroke, uncontrolled hypertension, peripheral vascular disease
Adverse effects	Generally well tolerated; nausea, somnolence, dry mouth (rare: liver enzyme elevation with older gepants)	Chest tightness, paresthesias, flushing, dizziness; risk of vasospasm; medication overuse headache
Drug interactions	CYP3A4 metabolism (care with inhibitors/inducers)	MAO-A inhibitors, SSRIs/SNRIs (risk of serotonin syndrome); other vasoconstrictors
Formulations	Oral tablets (ubrogepant, rimegepant), orally disintegrating tablets	Oral, intranasal, subcutaneous, orally disintegrating tablets
Dosing frequency	Can be repeated after 2 h (ubrogepant); rimegepant can be used every other day for prevention	Can repeat in 2 h; max daily and monthly use limits due to risk of overuse headache
Suitability	Good for patients with cardiovascular risk, poor tolerance to triptans, or contraindications to vasoconstrictors	Best for patients without cardiovascular disease needing rapid pain relief

Abbreviations: CAD, Coronary Artery Disease; MAO-A, Monoamine Oxidase; SSRIs/SNRIs, Selective Serotonin Reuptake Inhibitors/Serotonin–Norepinephrine Reuptake Inhibitors; CYP3A4 – Cytochrome P450 3A4.

Within this therapeutic class, distinct pharmacological and clinical profiles can be observed. Atogepant is the first gepant developed exclusively for preventive therapy, showing robust reductions in monthly migraine days in both episodic and chronic migraine. Ubrogepant, characterized by the shortest half-life, was the first oral gepant approved for the acute treatment of migraine, offering a well-tolerated alternative to triptans. Rimegepant remains unique as the only gepant with regulatory approval for both acute and preventive indications, bridging the gap between as-needed and continuous therapy. Its orally disintegrating tablet formulation further enhances patient convenience. Zavegepant, the first third-generation gepant and the only intranasally administered agent, provides rapid absorption and is particularly valuable for patients with nausea, vomiting, or rapid-onset attacks where oral administration is less suitable (for review see Table 3).

**TABLE 3:** Clinical and pharmacological comparison of atogepant, ubrogepant, rimegepant, and zavegepant in migraine treatment (based on (Dodick et al., 2019; Goadsby et al., 2020; Ailani et al., 2021b; Lipton et al., 2019; Scott, 2020b; AbbVie, 2023a; Croop et al., 2021a; ClinicalTrials.gov, 2025j; Lipton et al., 2023d; ClinicalTrials.gov, 2025k; Mullin et al., 2024))

Feature	Atogepant	Ubrogepant	Rimegepant	Zavegepant
Generation	2nd	2nd	2nd	3rd
Formulation	Oral tablet (10, 30, 60 mg)	Oral tablet (50, 100 mg)	Orally disintegrating tablet (75 mg ODT)	Intranasal spray (10 mg)
Dosing	Once daily (preventive use)	As needed for acute migraine	As needed for acute migraine; every other day for prevention	Single-dose spray for acute migraine
Onset of action (Tmax)	∼1–2 h	∼1–1.5 h	∼1.5 h	Rapid (minutes; intranasal)
Half-life (t½)	∼11 h	5–7 h	11 h	∼6–7 h
FDA approval	Sept 2021 (Qulipta®) – prevention of episodic and chronic migraine	Dec 2019 (Ubrelvy®) – acute treatment	Feb 2020 (Nurtec ODT®) – acute; May 2021 – prevention	2023 (Zavzpret™) – acute treatment
EU approval	Approved (Aquipta® - episodic and chronic prevention)	Not approved	Approved (VYDURA® -acute + prevention)	Under evaluation (not yet EU-approved)
Indications	Prevention only (episodic + chronic migraine)	Acute treatment only	Acute treatment and prevention (episodic migraine)	Acute treatment only
Key efficacy findings	↓ Monthly migraine days by ∼4–5 vs. placebo in chronic and episodic prevention trials	2 h pain freedom ∼19–21% vs. 12% placebo	Acute: 2 h pain freedom 21% vs. 11% placebo; Preventive: −4.3 vs. −3.5 MMDs	2 h pain freedom: 24% vs. 15% placebo
Safety profile	Common AEs: constipation, nausea, fatigue; generally well tolerated	Mild AEs: nausea, somnolence, dry mouth; no hepatotoxicity	Common AEs: nausea, urinary tract infection; well tolerated	Common AEs: dysgeusia (altered taste), nasal discomfort
Drug interactions	Substrate of CYP3A4; caution with strong inhibitors/inducers	CYP3A4 substrate; avoid strong inhibitors/inducers	CYP3A4 metabolism; avoid strong inhibitors/inducers	Minimal CYP metabolism; fewer interactions
Unique features	First gepant developed solely for preventive therapy	First gepant approved for acute attacks	Only gepant with dual acute + preventive indication	First intranasal gepant; rapid non-oral rescue
Clinical positioning	Daily preventive option, especially for patients intolerant to monoclonal antibodies	Oral alternative to triptans for acute attacks	Most versatile gepant (acute + prevention); convenient ODT	Rapid rescue therapy for acute migraine, especially in patients with nausea/vomiting

Abbreviations: Tmax, Time to Maximum Plasma Concentration; t½, Half-life; FDA, U.S., food and drug administration; EU, European Union; AEs, Adverse Events; CYP3A4, Cytochrome P450 3A4; ODT, Orally Disintegrating Tablet; MMDs, Monthly Migraine Days.

Taken together, gepants form a complementary therapeutic spectrum: atogepant as a daily preventive, ubrogepant and rimegepant as oral acute options (with rimegepant also approved for prevention), and zavegepant as a fast-acting, non-oral alternative. This diversity allows for individualized treatment strategies tailored to patient-specific needs and clinical profiles. Nevertheless, further studies are warranted to clarify the long-term safety of gepants, their potential preventive role beyond current indications, and their place in therapy in light of the still incompletely understood mechanisms of migraine initiation and progression.

## 6 Limitations and future directions

While gepants have demonstrated promising efficacy and tolerability across multiple phase 2 and 3 clinical trials, several important limitations remain. To date, most studies have been of relatively short duration (typically 12 weeks), leaving uncertainties regarding the long-term safety of continuous use, particularly with respect to rare or cumulative adverse events. Additionally, head-to-head comparative trials between gepants and established treatments such as triptans or monoclonal antibodies have not yet been conducted, limiting conclusions about their relative effectiveness in real-world settings.

Another important limitation is the lack of direct comparative trials among different gepants themselves, which would clarify their differential clinical positioning beyond pharmacokinetic and regulatory distinctions. Furthermore, while gepants appear to have a favorable hepatic safety profile compared with first-generation compounds, ongoing pharmacovigilance is warranted, particularly in patients with comorbidities or polypharmacy.

Future research should prioritize long-term extension studies, real-world observational data, and comparative effectiveness trials. Special attention should also be given to exploring the potential dual role of ubrogepant and atogepant in both acute and preventive therapy, as well as the utility of zavegepant in patients with rapid-onset or treatment-resistant migraine. Clarifying the exact mechanisms by which gepants exert their therapeutic effects, especially in relation to migraine initiation and progression, may also open avenues for next-generation therapies. In the future, it also seems possible to use gepants in combination with triptans to treat treatment-resistant migraine attacks (Visser et al., 1996). It is worth mentioning a recent study which, for the first time, compared the efficacy of different CGRP antagonists in the prevention of episodic migraine. Galcanezumab was not superior to rimegepant in reducing the number of migraine days per month by ≥ 50% from baseline (62% versus 61%, respectively; p = 0.70). Studies with gepants and monoclonal antibodies are still needed (Schwedt et al., 2024).

Taken together, gepants form a complementary therapeutic spectrum: atogepant as a daily preventive, ubrogepant and rimegepant as oral acute options (with rimegepant also approved for prevention), and zavegepant as a fast-acting, non-oral alternative. This diversity allows for individualized treatment strategies tailored to patient-specific needs and clinical profiles. Nevertheless, further studies are warranted to clarify the long-term safety of gepants, their potential preventive role beyond current indications, and their place in therapy in light of the still incompletely understood mechanisms of migraine initiation and progressions practical.

Together, these agents form a complementary therapeutic spectrum: atogepant as a daily preventive, ubrogepant and rimegepant as oral acute options (with rimegepant also preventive), and zavegepant as a fast-acting non-oral alternative. This diversity allows for highly individualized treatment strategies, aligning drug selection with patient-specific needs and clinical profiles.
